# Apolipoprotein E2 Genotype Is Associated with a 2-Fold Increase in the Incidence of Type 2 Diabetes Mellitus: Results from a Long-Term Observational Study

**DOI:** 10.1155/2019/1698610

**Published:** 2019-08-07

**Authors:** Cátia Santos-Ferreira, Rui Baptista, Manuel Oliveira-Santos, Regina Costa, José Pereira Moura, Lino Gonçalves

**Affiliations:** ^1^Department of Cardiology, Coimbra University Hospital Center, Coimbra, Portugal; ^2^iCBR, Faculty of Medicine, University of Coimbra, Portugal; ^3^Department of Internal Medicine, Coimbra University Hospital Center, Coimbra, Portugal; ^4^Faculty of Medicine, University of Coimbra, Portugal

## Abstract

**Background:**

The apolipoprotein E (APOE) polymorphisms are associated with cardiovascular (CV) disease, but its interaction with type 2 diabetes mellitus (T2DM) long-term incidence is unknown. We investigated the association between APOE genotype and long-term (i) CV events and (ii) T2DM incidence in a Southern European primary prevention cohort.

**Methods:**

We assessed individual APOE genotypes in a total of 436 patients followed at a lipid clinic, with a 15-year median follow-up time. We collected data on major CV events (CV death, myocardial infarction, and stroke) and T2DM development.

**Results:**

No differences were found regarding major CV event incidence among the different APOE genotypes. However, after excluding 39 patients with a prior history of T2DM, APOE2 carriers displayed a higher incidence of T2DM during follow-up (42.2%) than APOE3 (27.1%) and APOE4 (28.7%) carriers. The age-, sex-, triglycerides-, and statin usage-adjusted OR for T2DM incidence in APOE2 carriers was 1.8 (95%CI 1.1-2.9,* p*=0.03), compared with wild-type APOE3. To address the role of statins as a confounder, we analyzed T2DM incidence in statin-treated patients. Statin-treated APOE2 carriers also had a higher T2DM incidence (57.9%), in comparison with APOE3 homozygotes (31.6%) and APOE4 carriers (32.5%). After adjustment for confounding, APOE2 carriers on statins displayed a similar twofold increase in T2DM risk compared to APOE3 homozygotes (OR 2.1, 95%CI 1.1-4.0,* p*=0.03).

**Conclusion:**

Our findings suggest a twofold increase in T2DM incidence in APOE2 carriers. This may prompt for a specific glucose dysmetabolism follow-up that might be tailored on the APOE genotype.

## 1. Introduction

Cardiovascular (CV) disease remains the leading cause of morbidity and mortality in developed countries, despite consistent improvement in outcomes [1]. The CV risk is influenced by environmental and genetic factors, as apolipoprotein E (APOE) [2]. APOE, located in chromosome 19, is a polymorphic glycoprotein that plays a multifunctional role in lipid metabolism [3]. It is essential in the formation of chylomicrons, very low-density (VLDL) and high-density lipoproteins (HDL); and it is also involved in the transport of cholesterol from the peripheral tissues to the liver [4]. The APOE gene has three alleles (E2, E3, and E4) that produce 6 different genotypes (E2/2, E2/3, E2/4, E3/3, E3/4, and E4/4) [3]. The APOE locus has been identified as a susceptibility locus for coronary heart disease (CHD) for years, even though the results of epidemiologic studies examining this association are inconsistent [5–11].

Historically, APOE4 carriers have been described to suffer from a higher risk of (i) developing CHD, (ii) being submitted to coronary revascularization procedures, and (iii) dying from CHD [5–7]. More recently, while some data failed to reproduce such associations [8, 9], newer evidence supported the role of E4 allele as a risk factor for CHD [10, 11]. Additionally, controversy exists about these polymorphisms impact on type 2 diabetes mellitus (T2DM) prevalence [12]. Interestingly, the long-term incidence of T2DM according to the different APOE genotypes has never been evaluated in a prospective way. Our group has previously shown that APOE4 carriers were referred to a specialized lipid clinic at a younger age (44.2 ± 14.7 years) compared with non-APOE4 carriers (50.6 ± 13.8 years) (*p*<0.001) [13]. In this study, we aimed to investigate the impact of the APOE genotype on long-term (i) CV outcomes and (ii) T2DM incidence in a Southern European cohort of patients.

## 2. Material and Methods

### 2.1. Study Design and Population

We conducted a single-center study, prospectively including 691 consecutively admitted and followed in a tertiary hospital specialized dyslipidemia outpatient clinic between January 1994 and October 2007. All patients were referred to consultation by their primary care physician or by other specialists within the hospital due to markedly abnormal, difficult to control lipid profile or due to suspected familial dyslipidemia. No formal recommendations were given regarding which type of drugs should be used for reaching the lipid target. APOE genotypes were not taken into account when making therapeutic decisions regarding lipid-lowering drugs, as genotyping was performed solely with an investigational purpose [13]. Patients with familial hypercholesterolemia (HeFH), who were not genotyped and with a prior history of CV events, were excluded. The patients were followed for a median (interquartile range (IQR)) of 15 (12-17) years; all patients had more than 10 years of follow-up. Only 3 patients (0.7%) were lost to follow-up.

The study was approved by the local institutional board and all patients gave informed consent.

### 2.2. Risk Factors Assessment

Baseline, demographic, and clinical variables are collected, including age at referral, gender, T2DM, smoking status, and alcohol consumption. Diabetes was defined as fasting glucose ≥3.3 mmol.L^−1^ or use of hypoglycemic drugs. Hypertension was defined as systolic blood pressure (BP) ≥140mm Hg and/or diastolic BP ≥90 mmHg or current antihypertensive treatment. Current smoking (≥1 cigarette per day) and drinking alcohol (>2 units of alcoholic beverages per day) were defined. On the first consultation, several baseline laboratory variables were obtained, and a complete lipid profile was assayed by standard techniques in 12-h fasting blood samples, including total cholesterol (TC), HDL, LDL, triglycerides, apolipoprotein (apo) A and apoB, and lipoprotein (Lp) (a) levels [13].

### 2.3. DNA Extraction and APOE Genotyping

DNA was extracted from whole-blood specimens according to standard procedures. Genomic DNA from these samples was analyzed for APOE polymorphisms (rs7412 and rs429358) using polymerase chain reaction and reverse hybridization. Details of the DNA sequencing assessment of the APOE genotype have been published elsewhere [14]. ApoE concentrations were measured by nephelometry.

### 2.4. Definition of Events

All participants were followed up through record linkage with the national health registry and Health Data Platform (PDS). The primary endpoint was a composite of CV mortality, MI, and stroke. MI was defined as acute myocardial injury with clinical evidence of acute myocardial ischemia and with detection of a rise/or fall of cardiac troponin values with at least one value above 99th percentile upper reference limit and at least one of the following: (i) symptoms of myocardial ischemia; (ii) new ischemic ECG changes; (iii) development of pathological Q waves; (iv) imaging evidence of new loss of viable myocardium or new regional wall motion abnormality in a pattern consistent with an ischemic etiology; (v) identification of a coronary thrombus by angiography [15]. Stroke was defined as an acute episode of focal or global neurological dysfunction caused by brain, spinal cord, or retinal vascular injury as a result of hemorrhage or infarction [16]. CV death was defined as death resulting from an acute MI, sudden cardiac death, death due to heart failure, death due to stroke, death due to CV procedures, death due to CV hemorrhage, and death due to other CV causes [16]. The secondary endpoint was T2DM incidence.

### 2.5. Statistical Analysis

Allele frequencies were determined using the gene counting method. Hardy-Weinberg equilibrium for the distribution of the genotype was performed. Continuous variables were expressed as mean ± SD. Median and IQR were used if the distribution was not normal, assessed by the use of the Kolmogorov-Smirnov test. The one-way ANOVA for normal variables and the Kruskal Wallis test for nonnormal variables were used for comparisons among groups. Categorical variables were presented as percentages and were compared using chi-square or Fisher's exact test.

The number of patients in some individual genotype groups was too small to support group comparisons; therefore, and in a similar way to several other reports, we compared patients with one or more copies of the E4 allele (APOE4 carriers) or with one or more copies of the E2 allele (APOE2 carriers) to those without (APOE3 homozygotes) [9]. As in many other studies of this nature, eight subjects with the E4/2 genotype were excluded from the subsequent analyses because the E2 and E4 alleles are proposed to have opposite effects on CHD risk [9]. The CV risk was examined in relation to the APOE alleles first in an unadjusted model, followed by adjustment for age, sex, hypertension (yes or no), diabetes mellitus (yes or no), smoking status (current, nonsmoker), and statin therapy (yes or no). Participants were censored at the time of the first occurrence of CV event, death, or time of the last follow-up. The T2DM was also examined in relation to APOE alleles first in an unadjusted model, followed by adjustment for age, sex, statin therapy (yes or no), and triglycerides. Participants were censored at the time of the diagnosis of T2DM, death, or time of the last follow-up. All statistical analyses were performed using SPSS 24.0 (SPSS Inc., Chicago, Illinois, USA) with the level of significance set at* p*<0.05.

## 3. Results

### 3.1. Patient Population

We included 444 Caucasian patients (259 men and 185 women) of Southern European ancestry who were genotyped. The frequencies of E2, E3, and E4 alleles were 7.9, 78.5, and 13.6%, respectively (Table 1). Overall, after subjects with the E4/2 genotype were excluded, 283 of 436 patients were APOE3 homozygotes (64.9%), 102 were APOE4 carriers (23.4%), and 51 were APOE2 carriers (11.7%). The distribution of the APOE alleles was in Hardy-Weinberg equilibrium. No significant differences were found among gender regarding the distribution of the alleles.

The APOE groups were compared regarding demographic, clinical and laboratory variables (Tables 2 and 3). At baseline, no differences were found with regard to gender, weight, smoking habits, and alcohol consumption. Although the average blood pressure values were in the normal range among groups, hypertension was less prevalent in APOE4 carriers, but not reaching statistical significance (APOE4: 38.2% vs. APOE2: 52.9% and APOE3: 49.1%,* p*=0.11). In contrast, APOE2 carriers had a numerically higher prevalence of T2DM (APOE2: 11.8% vs. APOE3: 8.8% and APOE4: 7.8%,* p=*0.72).

**Figure 1:**
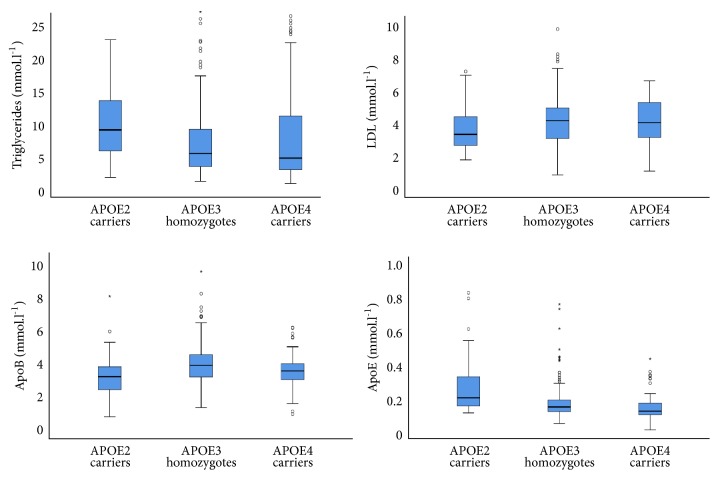
*Lipid profile by APOE genotype.* APOE: apolipoprotein E; LDL: low-density lipoprotein; Apo: apolipoprotein.

Regarding the lipid profile, LDL, triglycerides, ApoE, and ApoB differed significantly among the groups (Figure 1). APOE2 carriers had lower LDL (APOE2: 3.3 (2.6-4.5) vs. APOE3: 4.1 (3.0-4.9) and APOE4: 4.0 (3.0-5.3) mmol.L^−1^,* p*=0.04) and ApoB levels (APOE2: 3.1 (2.4-3.7) vs. APOE3: 3.8 (3.1-4.5) and APOE4: 3.5 (3.0-3.9) mmol.L^−1^,* p*<0.001). In contrast, triglycerides were markedly higher in APOE2 carriers compared to the remaining groups (APOE2: 8.7 (5.5-13.5) vs. APOE3: 5.2 (3.2-8.8) and APOE4: 4.5 (2.8-11.0) mmol.L^−1^,* p*<0.001). As expected, lower ApoE concentrations were found in APOE4 carriers (APOE4: 0.11 (0.09-0.16) vs. APOE2: 0.20 (0.15-0.33) and APOE3: 0.14 (0.11-0.18) mmol.L^−1^,* p*<0.001).

No differences were found regarding aspirin, angiotensin-converting enzyme inhibitors, angiotensin receptor blockers, beta-blockers, or calcium channel antagonists usage among groups. Regarding prior antidyslipidemic drug usage, statins were used more frequently in APOE3 homozygotes compared to APOE2 and APOE4 carriers (APOE3: 63.3%, higher than APOE2: 47.1%, and APOE4: 47.1%,* p*=0.005).

### 3.2. APOE Genotype and Long-Term CV Outcomes

**Figure 2:**
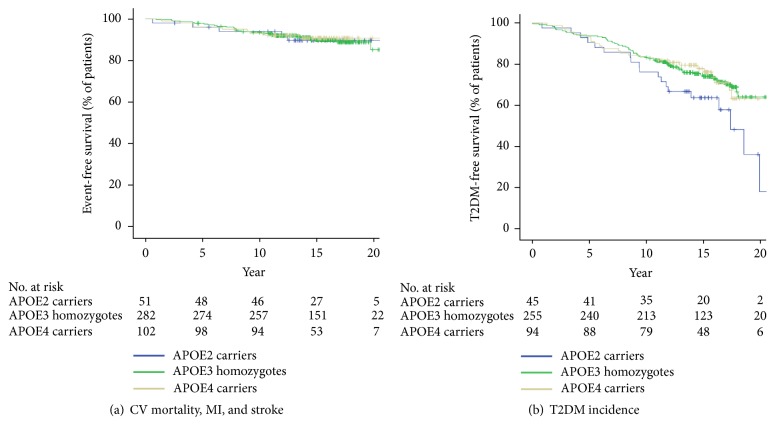
*Kaplan–Meier estimates of (a) CV mortality, MI and stroke and (b) T2DM incidence in the different APOE genotypes. *CV: cardiovascular; MI: myocardial infarction; T2DM: diabetes mellitus; APOE: apolipoprotein E.

The patients were followed for a median period of 15 (IQR 12-17) years. During this period, the primary endpoint occurred in 42 (9.6%) of the 436 subjects, including 19 (4.4%) MI and 16 (3.7%) strokes; 7 patients (1.6%) had CV death as their initial event. The median (IQR) time to the first event was 7 (4-11) years. No differences were found regarding the primary endpoint incidence at follow-up among APOE4 carriers (8.8%, unadjusted OR 0.9, 95%CI 0.4-1.9,* p*=0.82) or APOE2 carriers (9.8%, unadjusted OR 1.0, 95%CI 0.4-2.5,* p*=0.95) versus the wild-type APOE3 carriers (9.9%) (Figure 2(a)). Even after adjusting for age, sex, the prevalence of traditional CV risk factors (T2DM, hypertension, and smoking), and statin usage, there was no association between APOE polymorphisms and primary endpoint incidence (OR 0.9, 95%CI 0.3-3.1,* p*=0.89 and 1.3, 95%CI 0.5-3.3,* p*=0.62 for APOE2 and APOE4 carriers, respectively, when compared with APOE3 homozygotes).

### 3.3. APOE Genotype and T2DM Incidence

After noticing that the baseline prevalence of T2DM was numerically higher in APOE2 carriers, we determined T2DM incidence in the different APOE polymorphisms. All patients who had a prior history of T2DM (n = 39, 8.4%) were excluded and T2DM incidence at follow-up was determined in 390 patients (in 7 patients it was not possible to determine T2DM status at follow-up). T2DM was higher in APOE2 carriers (n = 19, 42.2%) than in APOE3 (n = 70, 27.1%) and APOE4 (n = 27, 28.7%) carriers. In the unadjusted analysis, T2DM incidence was higher in APOE2 carriers than APOE3 homozygotes (OR 1.8, 95%CI 1.1-2.9,* p*=0.03) (Figure 2(b)). After adjusting for age, sex, triglycerides, and statin use, we found a 1.8-fold incidence in APOE2 carriers (OR 1.8, 95%CI 1.1-3.1,* p*=0.03), compared with wild-type APOE3 carriers. Regarding APOE4 carriers, we did not find a significant difference in T2DM incidence compared to APOE3 (OR 1.2, 95%CI 0.8-1.8,* p*=0.47), even after adjusting for the same variables (OR 1.3, 95%CI 0.9-2.5,* p*=0.13).

As statins can play an important role as a confounder in T2DM incidence, we also estimated the proportion of patients on statins (n = 222) that developed T2DM. We found that APOE2 carriers under statin therapy had a numerically higher incidence of T2DM (57.9%) compared to APOE3 homozygotes (31.6%) and APOE4 carriers (32.5%). In contrast, patients without statins but under other lipid-lowering drugs (n = 35), no differences were found regarding T2DM incidence in the different APOE genotypes (APOE2: 44.4% vs. APOE3: 35.7% vs. APOE4: 58.3%,* p*=0.58). Statin-treated APOE2 carriers displayed a higher incidence of T2DM compared to APOE3 (age-, sex-, and triglycerides-adjusted OR 2.1, 95%CI 1.1-4.0,* p*=0.03). In contrast, statin-treated APOE4 carriers did not differ from APOE3 homozygotes (age-, sex-, and triglycerides-adjusted OR 1.3, 95%CI 0.7-2.4,* p*=0.42). Finally, no differences were found in the incidence of T2DM in statin-treated APOE2 carriers compared to nontreated (age-, sex-, and triglycerides-adjusted OR 1.9, 95%CI 0.6-5.7,* p*=0.27).

## 4. Discussion

In this study, we found that although APOE genotype was not a predictor of long-term CV events, there was a significant interaction between APOE genotypes and T2DM long-term incidence, with a higher incidence of long-term T2DM in APOE2 carriers.

Although the effect of APOE genotypes on CV outcomes is inconsistent in the literature, E4 allele seems to be associated with a slight increase of CHD [2, 5–7, 10, 11]. In our cohort, after adjusting for well-known CV risk factors, this association remained nonsignificant. The rate of events in our study was similar to the one observed in a cohort of 730 patients of the Baltimore Longitudinal Study of Aging with a mean follow-up time of 20 years for men and 13 years for women: 4.9% of patients had a MI and 1.0% had CV death [7]. However, contrary to our findings, this study observed that the APOE4 allele increased the risk of coronary events in men (RR 2.9, 95%CI 1.8-4.5,* p*<0.001), but not in women (RR 0.9, 95%CI 0.4-1.9,* p*=0.62) [7]. Among studies with shorter periods of follow-up, controversy remains whether APOE genotype is associated with coronary risk or not. A meta-analysis that included 17 studies observed a slightly higher CHD risk for APOE4 carriers (OR 1.06, 95%CI, 0.99-1.13) and a 20% lower risk in APOE2 carriers (OR 0.80, 95%CI, 0.70-0.90) [8]. Similarly, another meta-analysis that included 22 studies reported a higher risk of MI for APOE4 carriers (OR 1.20, 95%CI, 1.08-1.34) and a lower risk for APOE2 carriers (OR 0.79, 95%CI, 0.70-0.91) compared to wild-type APOE3 carriers [10]. In contrast, in the largest prospective cohort to date (n = 22,169), APOE genotype was not associated with CHD risk after controlling for a variety of CV risk factors, namely, lipid profile [9]. APOE4 may also be associated with an increased risk of cerebrovascular ischemic events [17, 18].

Although there are no data regarding T2DM incidence according to the different APOE polymorphisms, there are some estimates of T2DM prevalence, albeit inconsistent [12]. In a meta-analysis combining data from 30 independent cross-sectional studies (n = 13,620), APOE2 carriers had more commonly a prior history of T2DM (OR 1.18, 95%CI 1.02–1.35,* p*=0.023) [12]. In addition, recent genome-wide association studies have identified APOE as a novel T2DM susceptibility locus [19, 20]. In our study, the risk of developing T2DM on follow-up was significantly higher in APOE2 carriers (42.2%) than in wild-type APOE3 carriers (27.1%), after adjusting for age, sex, triglycerides, and statin use (OR 1.8, 95%CI 1.1-3.1,* p*=0.03). As the prevalence of T2DM in the Portuguese population aged between 65 and 75 is 23.8% [21], it is also of note the higher global incidence of T2DM in this cohort of patients. Probably we are facing a population with a high to very high 10-year risk of T2DM according to FINnish Diabetes Risk SCore (FINDRISC) [22].

The relationship between the APOE polymorphisms and T2DM is not clear. In the past, APOE4 was associated with a progressive increase in fasting glucose and T2DM, probably due to amyloid deposition within the islets of the pancreas [23]. The link between APOE2 and T2DM is also speculative. Although the majority of APOE2 homozygotes have normal or even lower plasma cholesterol levels, almost all APOE2 carriers have elevated triglycerides levels due to impaired hepatic clearance of triglyceride-rich lipoproteins [24]. A study using human APOE2 and APOE3 gene replacement mice showed that APOE2 mice had elevated fasting plasma triglyceride and insulin levels and displayed prolonged postprandial hyperlipidemia [25]. Importantly, impaired clearance of APOE2-containing triglyceride-rich lipoproteins from circulation leads to increased postprandial lipid uptake by leukocytes, promoting inflammation and chronic lipid deposition in adipose tissues [25]. The combination of elevated adiposity and inflammation increases the susceptibility to diet-induced obesity in APOE2 carriers and accelerates the development of hyperinsulinemia and ultimately T2DM [25]. Interestingly, patients with heterozygous HeFH have been reported to be less vulnerable to T2DM [26, 27]. Additionally, an inverse dose-dependent association was found in HeFH subjects with LDL receptor (LDLR) negative mutations who had a lower T2DM prevalence than carriers of defective LDLR or apolipoprotein B mutations [26]. A common pathway in HeFH and statin therapy—cellular cholesterol uptake—may play a role in the development of T2DM, perhaps because increased intracellular cholesterol levels are deleterious for pancreatic beta cell function [26]. However, if intracellular cholesterol uptake via the LDLR is exclusively involved, then it would only explain the hypothetical protection from diabetes in HeFH patients with genetic defects affecting LDLR uptake [27]. A different hypothesis is that the protection would be dependent on the high plasma LDL cholesterol concentrations observed in HeFH [27], disclosing the possible detrimental effect of normal or even lower levels of LDL cholesterol in APOE2 carriers.

These findings may have important clinical implications. The recognition of the higher risk of development of new-onset T2DM in APOE2 carriers may lead to strategies for earlier diagnosis of glucose intolerance with regular oral glucose loading tests and, consequently, earlier dietary and therapeutic interventions [1]. The treatment of dyslipidemia in these patients should also be reconsidered [28]. APOE polymorphisms not only influence on plasma lipid levels but also their response to statin therapy [29, 30]. Additionally, statin therapy is associated with a small, but significantly increased risk of T2DM development [31]. Pitavastatin appears to have a null effect on glucose metabolism after both short-and long-term therapy [32, 33] and probably should be favored over the others in patients at a higher risk of developing T2DM [34]. To balance the increase of plasma triglyceride levels and its deleterious effects in inflammation, a lower target of triglycerides levels may also be considered, following the available recommendations on T2DM patients with hypertriglyceridemia [28].

This study has several limitations. First, we cannot rule out that weak associations may have been undetected because of a lack of statistical power due to the limited sample size and the design of the study (observational). However, we have a long follow-up with a high number of events. Secondly, potential selection bias is another inherent limitation of this study. Our population was selected by primary care physicians for follow-up at a specialized lipid clinic and does not represent a cross-section of the population as a whole. Moreover, most of the patients were receiving lipid-lowering treatment at the time of inclusion, which may contribute to the absence of an association between APOE and CV risk, as statins may fade the different risk strata. Statins are also associated with a small, but significant increase in the risk of developing T2DM; however, the regression model was adjusted to this variable. Finally, we were also not able to adjust in our analysis for new prescription of statins or statin treatment duration during follow-up.

## 5. Conclusion

In summary, in a large, prospective, South European cohort of patients with a long-term follow-up, no interaction was found between APOE genotypes and CV outcomes. However, we found an age-, sex-, triglycerides-, and statin usage-adjusted 2-fold T2DM incidence in APOE2 carriers. This may prompt strategies for earlier diagnosis with regular oral glucose loading tests, better statin selection using less diabetogenic statins and eventually aim for lower lipid levels in this selected group.

## Figures and Tables

**Table 1:** Frequency of APOE genotype and APOE allele by gender.

	Total	Male	Female
	(N=444)	(N=259)	(N=185)
Genotype E2/2 – no. (%)	11 (2.5)	7 (2.7)	4 (2.2)
E3/2 – no. (%)	40 (9.0)	25 (9.7)	15 (8.1)
E 4/2 – no. (%)	8 (1.8)	4 (1.5)	4 (2.2)
E3/3 – no. (%)	283 (63.7)	163 (62.9)	120 (64.9)
E4/3 – no. (%)	91 (20.5)	54 (20.8)	37 (20.0)
E4/4 – no. (%)	11 (2.5)	6 (2.3)	5 (2.7)
Allele			
E2 – no. (%)	70 (7.9)	43 (8.3)	27 (7.3)
E3 – no. (%)	697 (78.5)	405 (78.2)	292 (78.9)
E4 – no. (%)	121 (13.6)	70 (13.5)	51 (13.8)

**Table 2:** Baseline characteristics of the 436 patients with different APOE alleles.

	APOE2 carriers	APOE3 homozygotes	APOE4 carriers	*p *value
	(N=51)	(N=283)	(N=102)	
Age – years	53±13	50±14	44±15	<0.001
Male – no. (%)	32 (62.7)	163 (57.6)	60 (58.4)	0.79
Weight – kg	79±9	77±14	76±14	0.51
SBP – mmHg	135 (125-150)	135 (122-146)	126 (115-140)	0.16
DBP – mmHg	88 (80-90)	80 (76-90)	80 (70-90)	0.09
TC – mmol.L^−1^	6.8 (5.7-8.5)	7.0 (6.0-8.2)	6.9 (5.9-7.9)	0.53
HDL cholesterol – mmol.L^−1^	1.1 (1.0-1.4)	1.2 (1.0-1.5)	1.2 (0.9-1.5)	0.46
LDL cholesterol – mmol.L^−1^	3.3 (2.6-4.5)	4.1 (3.0-4.9)	4.0 (3.0-5.3)	0.04
Lp(a) – mmol.L^−1^	0.36 (0.13-0.93)	0.39 (0.16-0.96)	0.36 (0.16-0.96)	0.94
ApoE – mmol.L^−1^	0.20 (0.15-0.33)	0.14 (0.11-0.18)	0.11 (0.09-0.16)	<0.001
ApoB – mmol.L^−1^	3.1 (2.4-3.7)	3.8 (3.1-4.5)	3.5 (3.0-3.9)	<0.001
ApoA – mmol.L^−1^	3.8 (3.2-4.4)	4.0 (3.5-4.6)	3.8 (3.2-4.4)	0.28
ApoB/ApoA	0.8 (0.6-1.0)	1.0 (0.7-1.2)	0.9 (.8-1.1)	0.10
Triglycerides – mmol.L^−1^	8.7 (5.5-13.5)	5.2 (3.2-8.8)	4.5 (2.8-11.0)	<0.001
Serum creatinine – *µ*mol.L^−1^	68.6 (68.6 -76.3)	68.6 (61.0-76.3)	68.6 (61.0-76.3)	0.65

APOE: apolipoprotein E; SBP: systolic blood pressure; DBP: diastolic blood pressure; TC: total cholesterol; LDL: low-density lipoprotein; HDL: high-density lipoprotein; Lp(a): lipoprotein (a); Apo: apolipoprotein.

**Table 3:** Baseline prior history and medication of the 436 patients with different APOE alleles.

	APOE2 carriers	APOE3 homozygotes	APOE4 carriers	*p *value
	(N=51)	(N=283)	(N=102)	
Prior history				
HTA – no. (%)	27 (52.9)	139 (49.1)	39 (38.2)	0.11
T2DM – no. (%)	6 (11.8)	25 (8.8)	8 (7.8)	0.72
Current smokers – no. (%)	10 (25.0)	49 (22.2)	12 (15.0)	0.32
Alcohol consumption – no. (%)	13 (25.5)	65 (23)	12 (11.8)	0.37
Prior medication				
Aspirin– no. (%)	17 (33.3)	85 (30.0)	23 (22.5)	0.26
Beta-blockers– no. (%)	7 (13.7)	45 (15.9)	11 (10.8)	0.45
ACEi – no. (%)	12 (23.5)	75 (26.5)	17 (16.7)	0.14
ARB – no. (%)	7 (13.7)	38 (13.4)	14 (13.7)	0.99
Calcium antagonist – no. (%)	8 (15.7)	36 (12.7)	13 (12.7)	0.84
Statins – no. (%)	24 (47.1)	179 (63.3)	48 (47.1)	0.005
Ezetimibe – no. (%)	7 (13.7)	34 (12.0)	6 (5.9)	0.18
Fibrates – no. (%)	15 (29.4)	47 (16.6)	24 (23.5)	0.06
Niacin/omega-3 fatty acids – no. (%)	1 (2.0)	10 (3.5)	1 (1.0)	0.48

HTA: hypertension; T2DM: type 2 diabetes *mellitus*; ACE: angiotensin-converting enzyme inhibitor; ARB: angiotensin receptor blocker.

## Data Availability

The data used to support the findings of this study are included within the article.
